# Molecular Determinants of Survival Motor Neuron (SMN) Protein Cleavage by the Calcium-Activated Protease, Calpain

**DOI:** 10.1371/journal.pone.0015769

**Published:** 2010-12-30

**Authors:** Jennifer L. Fuentes, Molly S. Strayer, A. Gregory Matera

**Affiliations:** Program in Molecular Biology and Biotechnology, Departments of Biology and Genetics, Lineberger Comprehensive Cancer Center, University of North Carolina, Chapel Hill, North Carolina, United States of America; Brunel University, United Kingdom

## Abstract

Spinal muscular atrophy (SMA) is a leading genetic cause of childhood mortality, caused by reduced levels of survival motor neuron (SMN) protein. SMN functions as part of a large complex in the biogenesis of small nuclear ribonucleoproteins (snRNPs). It is not clear if defects in snRNP biogenesis cause SMA or if loss of some tissue-specific function causes disease. We recently demonstrated that the SMN complex localizes to the Z-discs of skeletal and cardiac muscle sarcomeres, and that SMN is a proteolytic target of calpain. Calpains are implicated in muscle and neurodegenerative disorders, although their relationship to SMA is unclear. Using mass spectrometry, we identified two adjacent calpain cleavage sites in SMN, S192 and F193. Deletion of small motifs in the region surrounding these sites inhibited cleavage. Patient-derived SMA mutations within SMN reduced calpain cleavage. SMN(D44V), reported to impair Gemin2 binding and amino-terminal SMN association, drastically inhibited cleavage, suggesting a role for these interactions in regulating calpain cleavage. Deletion of A188, a residue mutated in SMA type I (A188S), abrogated calpain cleavage, highlighting the importance of this region. Conversely, SMA mutations that interfere with self-oligomerization of SMN, Y272C and SMNΔ7, had no effect on cleavage. Removal of the recently-identified SMN degron (Δ268-294) resulted in increased calpain sensitivity, suggesting that the C-terminus of SMN is important in dictating availability of the cleavage site. Investigation into the spatial determinants of SMN cleavage revealed that endogenous calpains can cleave cytosolic, but not nuclear, SMN. Collectively, the results provide insight into a novel aspect of the post-translation regulation of SMN.

## Introduction

Spinal Muscular Atrophy (SMA) is an autosomal recessive disorder and a leading genetic cause of childhood mortality [1], [2], [3]. SMA falls into three clinical classes: types I, II and III, based on the age of disease onset and phenotypic severity. It is characterized by a loss of lower spinal motor neurons and atrophy of the trunk and proximal limb muscles [4], [5]. The locus responsible for SMA was mapped to chromosome 5q13 [6], [7]. In humans, there are two genes, *SMN1* (telomeric) and *SMN2* (centromeric), located near each other at this locus [8]. The protein coding sequences of *SMN1* and *SMN2* are predicted to be identical, as *SMN2* differs from *SMN1* by only five nucleotides [9], [10]. In *SMN2*, a single C to T transition in exon 7 leads to aberrant splicing, producing primarily transcripts lacking exon 7 (SMNΔ7) [11], [12]. The resultant SMNΔ7 protein is not fully functional and is less stable than full-length SMN [13], [14], [15], [16]. The severity of SMA is inversely proportional to *SMN2* copy number. This is due to the ability of *SMN2* to produce low levels (∼10%) of full-length SMN protein [17], [18]. Over 96% of SMA patients have homozygous mutations (deletion, rearrangement, or point mutation) in *SMN1*, however they retain at least one copy of *SMN2* [8], . These findings suggest that *SMN2* partially rescues the lethal *SMN1* loss-of-function phenotype, a hypothesis that has been substantiated by mouse models of SMA [20], [21].

SMN is thought to be involved in both tissue-specific and cell-essential functions. While global functions of SMN include the biogenesis of the small nuclear ribonucleoproteins (snRNPs) that carry out pre-mRNA splicing [22], [23], the putative tissue-specific-functions include axonal mRNA transport, neurite outgrowth, neuromuscular junction (NMJ) formation, myoblast fusion and myofibril integrity [24], [25], [26], [27], [28], [29]. The most well-characterized function of SMN is its role in snRNP biogenesis [30], [31]. During snRNP biogenesis SMN primarily associates with eight proteins, Gemins 2-8 and UNRIP/STRAP, to form the “SMN complex.” Following SMN-assisted RNP assembly, spliceosomal snRNPs are imported into the nucleus where they are further modified and remodeled in distinct nuclear subdomains, termed Cajal bodies (CBs). The snRNPs are subsequently released from the SMN complex and transit to interchromatin granule clusters [32]. It is currently unclear whether defective snRNP assembly and subsequent splicing of genes in motoneurons is responsible for SMA or if deficiencies in other tissue-specific functions of SMN cause the disease [33].

We previously demonstrated that the SMN complex localizes to both skeletal and cardiac myofibril Z-discs and interacts with α-actinin, an actin crosslinking protein [26], [34]. Treatment of skeletal myofibrils with exogenous calpain protease releases SMN from the sarcomere, identifying it as a calpain substrate. SMN is a proteolytic target of calpain, even when present in the native SMN complex [34]. Calpains are calcium-activated neutral cysteine proteases that are involved in numerous cellular processes, including myogenesis, muscle remodeling, and synaptic function (reviewed in [35], [36], [37], [38], [39], [40]). Calpains typically perform limited cleavage of their substrates, regulating their activity. Fourteen distinct calpains have been identified in humans, however the best characterized are the ubiquitous Calpain1 (µ-Calpain) and Calpain2 (m-Calpain). These large subunits (∼80 kDa) form heterodimers with a common small (∼28 kDa) regulatory subunit, called Calpain4. Calpains 1 and 2 are activated by micro- and milli-molar levels of calcium, respectively, and are inhibited *in vivo* by the protein calpastatin. Currently, it is unclear how the calpain-calpastatin system is regulated *in vivo*, however several possible modes of regulation have been proposed, such as local calcium transients, differential localization, post-translational modifications, and membrane association [37], [41], [42].

Calpains have been implicated in several muscle and neurodenerative disorders, including limb girdle muscular dystrophy type 2A (LGMD2A) [43], muscle cachexia [44], amylotrophic lateral sclerosis (ALS), multiple sclerosis (MS), Alzheimer's disease, Parkinson's disease, Huntington's disease, cerebral ischemia and prion-related encephalopathy [45]. Whether calpains play a role in SMA is not known. To further characterize the relationship between calpain and SMN, we characterized several determinants of cleavage activity. *In vitro* peptide mapping showed that Calpain1 cleaves SMN after residues S192 or F193, proximal to a proline-rich region; we determined that residues within a nearby PEST motif are important for this cleavage. Calpain was blocked by overexpression of calpastatin, but not by a D252A mutation, which reportedly blocks caspase cleavage of SMN [46]. Several SMA patient mutations residing in the N-terminus revealed a reduction in calpain susceptibility. One mutation, D44V, reported to inhibit Gemin2 binding [47], blocked calpain cleavage almost entirely. SMA mutations that affect the self-oligomerization properties of SMN, such as Y272C and SMNΔ7, had no major effect on cleavage, whereas increased calpain cleavage was observed by removal of the recently identified SMN degron (Δ268-294) [48]. Interestingly, an uncharacterized SMA mutation residing near the calpain cleavage sites, A188S, modestly reduced cleavage, and its deletion drastically impaired it, suggesting that this region is important for calpain cleavage. Finally, we determined that SMN is cleaved by cytosolic, but not nuclear calpains, suggesting a possible role for calpain in cytoplasmic regulation of SMN.

## Materials and Methods

### Cell culture, transfection, and DNA constructs

U2-OS osteosarcoma cells (American Type Culture Collection) were grown in DMEM supplemented with 10% fetal bovine serum, penicillin, and streptomycin at 37°C under 5% CO_2_. Transient transfection of plasmid DNA was performed using Effectene® transfection reagent, per manufacturer's instructions (Qiagen). Cells were harvested 24–36 hrs. post-transfection. Construct pEGFPC3-1-SMN was cloned by PCR amplification of h*SMN1* from previously constructed GFP-SMN* [49]. The PCR product was cloned into pCR®II-TOPO® (Invitrogen), per manufacturer's instructions, to create pCRII-TOPO-SMN, and subcloned into pEGFP-C3 (Clontech) using BglII and SalI sites. The plasmid used to express and copurify HIS_6_SMN/GST-Gemin2 heterodimers was created by first amplifying h*Gemin2* by PCR from pcDNA3-Flag-Gemin2 [50]. The PCR product was cloned into pCR®II-TOPO® (pCRII-TOPO-Gemin2) and the h*Gemin2* insert was digested with BglII and EcoRI enzymes and ligated to BamHI and EcoRI digested pGEX-3X (GE Healthcare) (pGEX3X-Gemin2). GST-Gemin2 was amplified by PCR and subcloned into pCDFDuet-1 (Novagen) using NdeI and XhoI sites (pCDFDuet1-GSTGemin2). Finally, *hSMN1* was digested with BglII and SalI enzymes from PCRII-TOPO-SMN and subcloned into BamHI and SalI digested pCDFDuet1-GSTGemin2 (pCDFDuet1-HIS_6_SMN-GSTGemin2). All deletions and point mutations in this study were created in pEGFPC3-1-SMN, by Quikchange® site-directed mutagenesis, per manufacturer's instructions (Stratagene). Primer sequences for all cloning and mutagenesis are available upon request. GFP-hCalpastatin and HA-hCalpastatin containing plasmids were kind gifts from Dr. Francesca Demarchi [51].

### Cell-free calpain assays

To prepare lysate, cell pellets were resuspended in ice-cold gentle binding buffer (50 mM Tris-HCl, pH 7.5, 200 mM NaCl, 0.2 mM EDTA, 0.05% NP-40) lacking protease inhibitors and pushed 10 times through a syringe fitted with a 25.5 gauge needle. The lysate was centrifuged at 14,000 RPM for 5 min. at 4°C. The total protein concentration of the supernatant was determined by Bradford assay using BSA as a standard [52]. Calpain assays were performed using 30 µg of total protein in a total reaction volume of 20 µL. Cleavage by endogenous calpains was activated by the addition of 1 mM CaCl_2_. Where indicated, exogenous Calpain1 (porcine erythrocytes, Calbiochem) and 1 mM CaCl_2_ were added. Calpain inhibitors N-acetyl-leucyl-leucyl-norleucinal, ALLN (Calbiochem) (inhibited cleavage at both 10 µM or 1 mM), and EGTA (4 mM) were added prior to the addition of calcium or exogenous Calpain1. Reactions were incubated for 15 min. (10 min. for reactions used for quantification) at 30°C and terminated by the addition of 5X SDS sample buffer (250 mM Tris-HCl, pH 6.8, 10% SDS, 50% glycerol, 500 mM DTT, 0.1% bromophenol blue), and heating at 100°C for 5 min.

### Cell fractionation

U2-OS cells were harvested and fractionated using the NE-PER® nuclear and cytoplasmic extraction reagents, per manufacturer's instructions, in the absence of protease inhibitors (Thermo Scientific). The nuclear lysates were dialyzed at 4°C in gentle binding buffer. Total protein concentration in the lysates was determined by Micro BCA™ Protein assay (Thermo Scientific). Calpain cleavage assays utilizing cytoplasmic and nuclear lysates were performed as described above.

### Western analysis and quantification of calpain cleavage

Proteins were separated by SDS-PAGE and transferred onto nitrocellulose (Whatman). Mouse monoclonal antibodies recognizing either the N-terminus (clone 8, BD Biosciences, 1∶10,000) or the C-terminus (9F2, L. Pellizzoni, 1∶10) of SMN were used. Rabbit polyclonal antibodies recognizing GAPDH (IMGENEX, 1∶4,000) or GFP (Invitrogen 1∶2,000) were also used. The appropriate secondary antibodies conjugated to HRP (Thermo Scientific, 1∶5,000-10,000) were used to obtain representative film images. To determine relative calpain susceptibilities of GFP-SMN constructs, quantification of full-length and N-terminal cleavage products was performed. While detectable by chemiluminescence, the C-terminal SMN cleavage product was not readily detected by fluorometry and thus was not used for quantification. The N-terminal SMN antibody, followed by a secondary antibody conjugated to Cy3 (GE Healthcare, 1∶4000) was used for quantification of calpain cleavage by fluorometry using unsaturated digital scans performed on a Typhoon Trio+ Variable Mode Imager (GE Healthcare). The integrated density of the full-length EGFP-SMN and the N-terminal cleavage product were quantified using ImageJ (http://rsbweb.nih.gov/ij/). Background signal was subtracted using the default rolling ball parameters. The percent cleavage of EGFP-SMN for each reaction was calculated by dividing the integrated density of the N-terminal cleavage fragment over the sum of the integrated density of the two bands. The average fraction cleaved was determined from six independent cell-free calpain assays.

### Purification and calpain cleavage of SMN/Gemin2 heterodimers

The pCDFDuet1-HIS_6_SMN-GSTGemin2 construct was transformed into BL21 Star™ (DE3) *E. coli* (Invitrogen). Cells were grown in 2 L LB containing streptomycin (50 µg/ml) at 37°C and induced with 1 mM IPTG (ACROS) at 30°C for 4 hr. Cells were harvested by centrifugation at 4°C for 10 min. at 3,500 RPM (F10S-6x500y rotor, Thermo Scientific) and resuspended in PBS containing protease inhibitors (Roche). Cells were lysed by sonication and incubated with 1% Triton X-100 for 30 min. at 4°C. Lysate was clarified by centrifugation at 4°C for 15 min. at 10,000 RPM (SLA-600 rotor, Sorvall). Clarified lysate was mixed with 600 µl bed volume Glutathione Sepharose™4B beads (GE Healthcare) at 4°C for 3 hr. Protein bound beads were washed extensively with PBS +1% Triton X-100 and subsequently PBS +0.1% Triton X-100, and stored O/N at −20°C in PBS +75% glycerol +0.1% Triton-X100. *In vitro* cleavage of HIS_6_SMN/GST-Gemin2 was performed by first equilibrating the protein bound beads in RSB100 + Ca^2+^ buffer (10 mM Tris-HCl, pH 7.5, 2.5 mM MgCl_2_, 100 mM NaCl, 0.1% NP-40, 1 mM CaCl_2_) by extensive washing. Equal volumes of protein-bound beads were then shaken (500 RPM) in the absence or presence of Calpain1 (1U or 2U) (Calbiochem) in 40 µl reactions for 1 hr. at 30°C. Reactions were terminated by adding 1% SDS and heating at 100°C for 10 min. The volume was increased to 200 µl with H_2_O and the samples were vortexed to further elute the proteins. The beads were pelleted by centrifugation and the supernatants were reduced in volume to 40 µl by vacuum. Proteins were reduced by incubating with 10 mM DTT (Fisher Scientific) at 50°C for 15 min. and subsequently alkylated by incubating with 50 mM iodoacetamide (Sigma) at room temperature for 30 min. in the dark.

### Peptide fingerprint analysis

HIS_6_SMN/GST-Gemin2 heterodimers were cleaved with Calpain1, as described above, and samples were mixed with 5X SDS sample buffer and proteins were separated by SDS-PAGE. The gel was stained with GelCode® Blue Stain, per manufacturer's instructions (Thermo Scientific), and submitted to the UNC Michael Hooker Proteomics Center for analysis. Individual gel bands were then manually excised and subjected to overnight-automated digestion with sequencing grade modified trypsin (Promega) on a ProGest Digestor (Genomic Solutions) at 37°C. Resultant peptides were lyophilized and re-dissolved in 5 uL of 50% methanol/0.1% trifluoroacetic acid (TFA). Peptides were spotted onto a MALDI target plate with an equal volume of α-cyano-4-hydroxycinnamic acid matrix solution and allowed to air dry. Mass spectrometry was carried out on a 4800 Plus MALDI TOF/TOF Analyzer (Applied Biosystems). Peptides were scanned in positive reflector mode over the mass range 700–4000 m/z, with internal calibration against trypsin peaks 842.51 and 2211.105 m/z. The forty most intense peptides were automatically selected for MS/MS analysis. Peptide mass and corresponding MS/MS fragmentation information for each sample were searched against the HIS_6_-SMN1 protein sequence using Mascot (Matrix Science) and GPS Explorer (Applied Biosystems).

## Results

### Exogenous and endogenous calpains cleave SMN to produce distinct cleavage products

**Figure 1:**
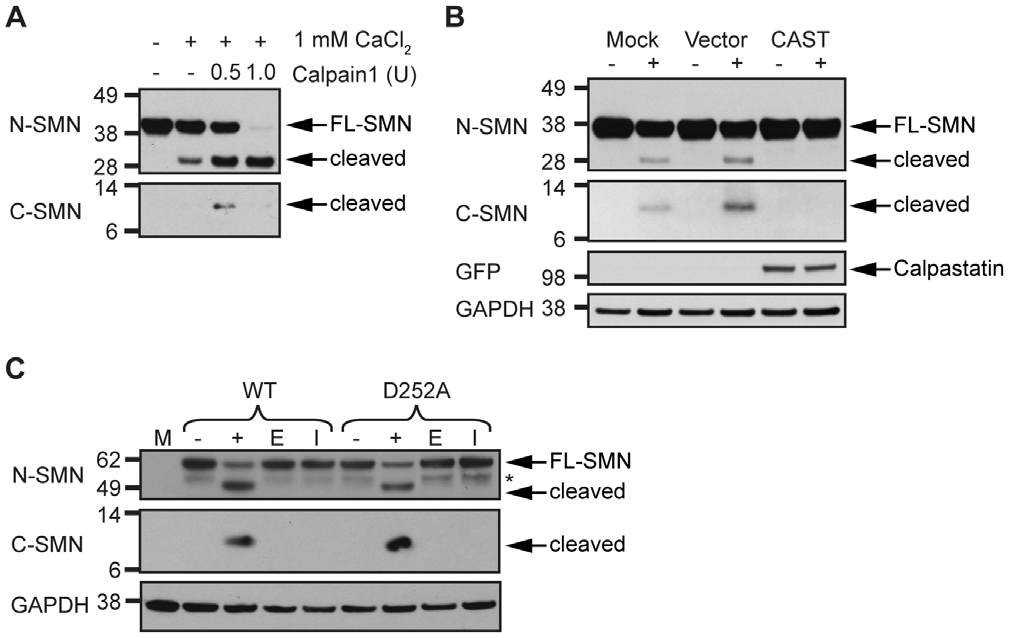
Western analysis of calpain assays detects two SMN cleavage products. (A) 1 mM CaCl_2_ and the indicated units of Calpain1 were incubated with U2-OS cell lysates. 30 µg total protein was used in each reaction. Both N-terminal and C-terminal cleavage products were observed with the indicated SMN antibodies (left). (B) Cells were mock transfected or transfected with either EGFP empty vector or EGFP-Calpastatin (CAST). Lysates were incubated in the absence (-) or presence (+) of 1 mM CaCl_2_ to activate SMN cleavage by endogenous calpains. Overexpression of calpastatin blocked calpain cleavage of SMN. (C) Cells were transfected with either EGFP-SMN or EGFP-SMN(D252A) and 1 mM CaCl_2_ (+, E, I) was added to the lysates. Where indicated, calpain cleavage was inhibited by addition of EGTA (E) or ALLN (I). Full-length GFP-SMN and cleavage products were detected by Western analysis using either N- or C-terminal SMN antibodies. As expected, the mock-transfected sample (M) did not contain GFP-tagged proteins. GAPDH was used as a loading control. *In the absence of calpain activation and protease inhibitors EGFP-SMN was subject to unknown protease(s), unrelated to calpains. Calpain cleavage products of EGFP-SMN that correlated to those observed upon calpain cleavage of endogenous SMN were studied.

We previously demonstrated that SMN is a target of calpain. Calpain cleavage of both native and recombinant SMN complexes leads to production of N- and C-terminal cleavage products. However, we had been unable to detect the C-terminal cleavage product in cell-free cleavage assays using HeLa cell lysates [34]. Subsequently, we found that addition of exogenous calpain in cell-free cleavage assays using U2-OS osteosarcoma cells results in a clearly detectable C-terminal cleavage product. Increased levels of exogenous calpain can lead to further cleavage and proteolysis of SMN (Fig. 1A), however whether this degree of proteolysis occurs *in vivo* is unknown. To confirm that calpains are responsible for the observed cleavage, we transiently expressed the calpain inhibitor calpastatin into U2-OS cells and performed cell-free cleavage assays. While several peptidyl inhibitors are commonly used to inhibit calpain, their inhibition is not limited to calpain proteases [53]. We therefore expressed calpastatin, which is the only known specific endogenous inhibitor of calpain [54]. Although the assay was not as robust (see Methods), we observed the C-terminal cleavage product upon treatment with calcium alone, demonstrating that cleavage by endogenous calpains also produces both cleavage products (Fig. 1B). Considering the amount of full-length SMN remaining after the addition of calcium, it is likely that only a small population of SMN proteins interact with endogenous calpains under these experimental conditions. Overexpression of GFP-Calpastatin or HA-Calpastatin blocked cleavage of SMN (Fig. 1B and data not shown), confirming that calpain is responsible for the calcium-activated cleavage of SMN *in vivo*. These results suggest that cleavage of SMN by calpain, and/or the stability of the cleavage products, may vary among cell types. Considering that calpains are generally thought to be regulatory proteases versus degradative ones [37], the detection of the cleavage products opens up the possibility that they may be stable enough to function in the cell. Notably, overexpression of the SMN C-terminus (amino acids 235-294) is reportedly sufficient to rescue neurite outgrowth [27], further supporting this notion.

### Calpain cleavage of SMN is distinct from caspase cleavage of SMN

Caspases and calpains are cysteine proteases involved in apoptosis, although the role of calpains in this process is not well defined [55]. The two protease families regulate each other directly, as well as through cleavage of the calpain inhibitor, calpastatin [56], [57], [58]. Virus-induced apoptosis and neuronal injury produce an N-terminal SMN cleavage product (∼29 kDa) and mutation of a predicted caspase cleavage site, D252A, blocks this cleavage [46]. SMN cleavage products, presumably due to caspase cleavage, are also generated in PC12 cells after deprivation of trophic support [59], further suggesting SMN is cleaved during apoptosis. To determine if the cleavage products we observed were different from the reported caspase cleavage product, we assayed the cleavage susceptibility of SMN(D252A) in cell-free cleavage assays. EGFP-SMN and EGFP-SMN(D252A) were transiently expressed in U2-OS cells, and cell lysates were incubated in the absence or presence of CaCl_2_ to activate endogenous calpains. Western analysis performed with antibodies that recognize either the N- or C-terminus of SMN showed that both WT and mutant SMN proteins were cleaved by calpain, and cleavage was blocked by pre-incubation of lysates with a calcium chelator (EGTA) or with a calpain inhibitor (ALLN, N-acetyl-Leu-Leu-norleucinal) [53] (Fig. 1C). These results indicate that the calpain cleavage site in SMN is distinct from the previously reported caspase cleavage site.

### Identifying sequence determinants of SMN cleavage

**Figure 2:**
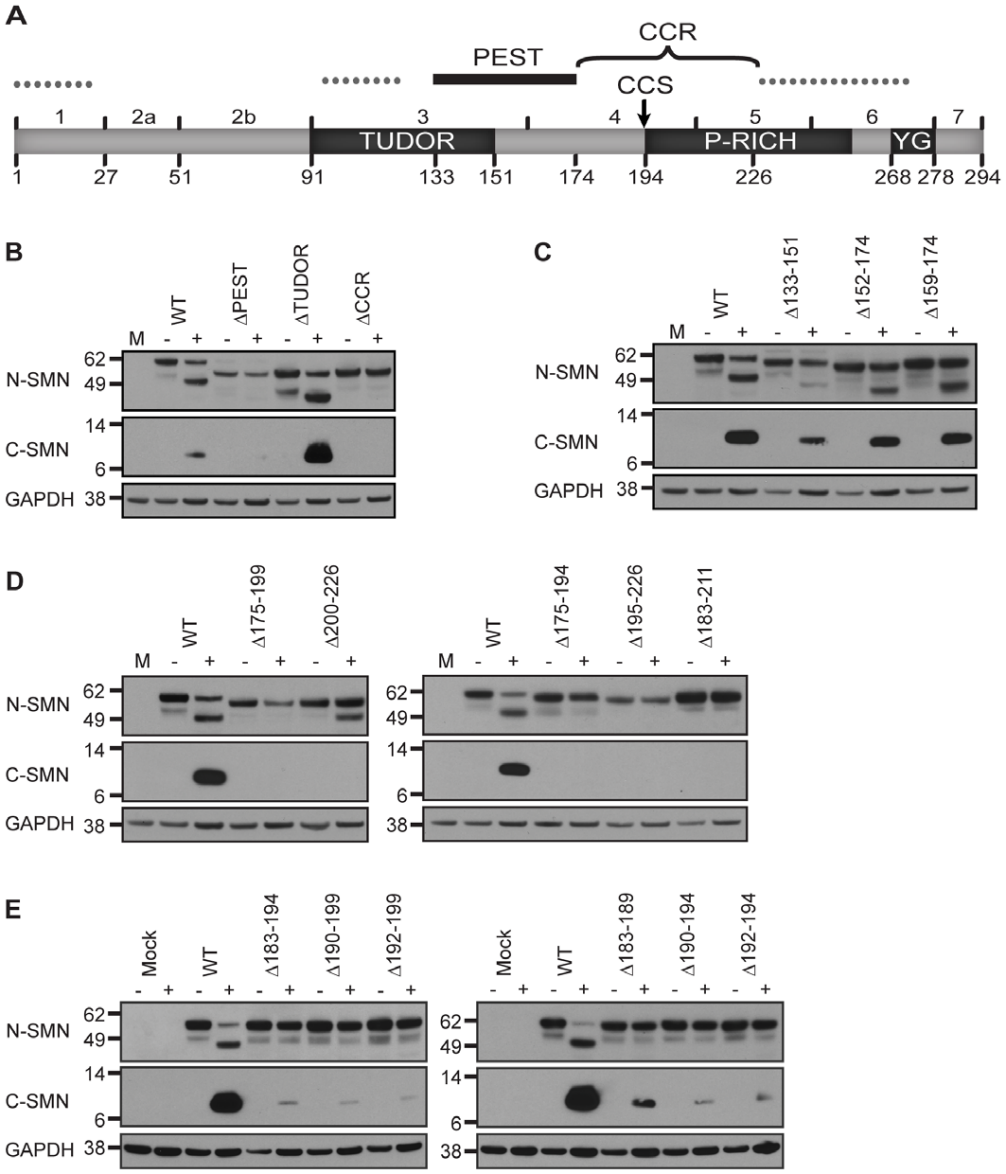
Sequence determinants of calpain cleavage of SMN. (A) Schematic of SMN protein, showing relevant domains and amino acids. The Tudor domain, proline-rich (P-rich) region, and YG box are labeled. Solid and dotted lines indicate the strong and weak PEST motifs, respectively. The calpain cleavage region (CCR) and mapped calpain cleavage site (CCS) are labeled. (B-F) Internal deletions were created in EGFP-SMN and transiently expressed in U2-OS cells. Endogenous calpain cleavage assays and subsequent Western analysis was performed to determine calpain cleavage susceptibility. Full-length GFP-SMN and cleavage products were detected using N- or C-terminal SMN antibodies. (B) The PEST motif and CCR are necessary for calpain cleavage, whereas the Tudor domain is dispensable. (C) Smaller deletions within the PEST domain allow for calpain cleavage. The entire PEST motif is not necessary for calpain cleavage. (D–E) Sequence determinants of calpain within the CCR. The CCR was progressively refined within residues 183–189 and 192–194.

Calpains recognize the tertiary structures of their substrates, and although there is no consensus recognition sequence, certain amino acids are preferred at the scissile peptide bond [60], [61], [62], [63]. In addition, the presence of PEST motifs, initially identified in short-lived proteins, often indicate the presence of nearby calpain proteolytic sites [64], [65]. PEST domains are regions rich in proline (P), glutamic acid (E), serine (S), and threonine (T) and can be computationally predicted. Analysis of SMN (http://mobyle.pasteur.fr/cgi-bin/portal.py?form=epestfind) reveals that it contains one strong (aa133-174), and three weak PEST (aa1-22, aa97-119, aa227-273) motifs (Fig. 2A). The strong PEST motif partially overlaps with the conserved Tudor domain (Fig. 2A), which interacts with RG-rich domains, such as those found on Sm proteins and the Cajal body marker protein, Coilin [66], [67], [68], [69], [70]. Considering the size of the SMN cleavage products (∼28 and ∼10 kDa), their differential reactivity to antibodies against the N- or C-terminus of SMN, and that calpain protease sites can reside within or adjacent to PEST motifs [64], [65], we predicted that sequences within *SMN* exons 4 or 5 contain the calpain cleavage site. This predicted calpain cleavage region (CCR) is downstream of the strong PEST motif and overlaps with the proline-rich region (Fig. 2A) that was shown to interact with the actin-binding protein, profilin [71], [72].

To identify amino acids in SMN that are important for calpain cleavage, we created constructs containing internal deletions in EGFP-SMN and tested their susceptibility to calpain cleavage in cell-free assays. Deletions targeted the Tudor domain (aa91-151), the strong PEST motif (aa133-174), and the CCR (aa175-226). The results demonstrate that the PEST motif and CCR are necessary for calpain cleavage, whereas the Tudor domain is dispensable (Fig. 2B). To determine if a smaller region of the PEST motif is sufficient to direct cleavage, we made smaller internal truncations. These deletions overlapped with the Tudor domain (aa133-151), with exon 3 (aa152-174), or did not overlap with either region (aa159-174). Smaller deletions within the PEST motif failed to block calpain cleavage of SMN (Fig. 2C), suggesting that the entire PEST motif is not necessary to direct calpain cleavage.

Analysis of smaller internal deletions in the CCR showed that deletion of amino acids 175-199, 175-194, 195-226, or 183-211 all blocked the calpain cleavage of SMN, whereas deletion of residues 200–226 did not. The C-terminal cleavage product of EGFP-SMNΔ200-226 was not detected by the anti C-terminal SMN antibody (Fig. 2D). This could result from deletion of the antibody epitope (which resides within aa188-268, L. Pellizzoni, personal communication), or from destabilization of the C-terminal product. Thus, the CCR could be narrowed down to amino acids 183-194; additional mutations within this twelve amino acid window substantially blocked calpain cleavage. As summarized in Fig. 2E, the smallest, non-overlapping deletions that inhibited cleavage were residues 183–189 (IKPKSAP), and 192-194 (SFL). Note that several of the deletions removed a stretch of five proline residues (P195-P199) in proline-rich region (Fig. 2A–E). Considering the importance of proline in protein secondary structures, we assayed whether mutation of these residues affected cleavage. However, we found that deletion or substitution by alanine or glycine residues did not block calpain cleavage (Fig. S1), suggesting that these putative structural changes were not significant enough to block cleavage.

### Mapping the calpain cleavage site

**Figure 3:**
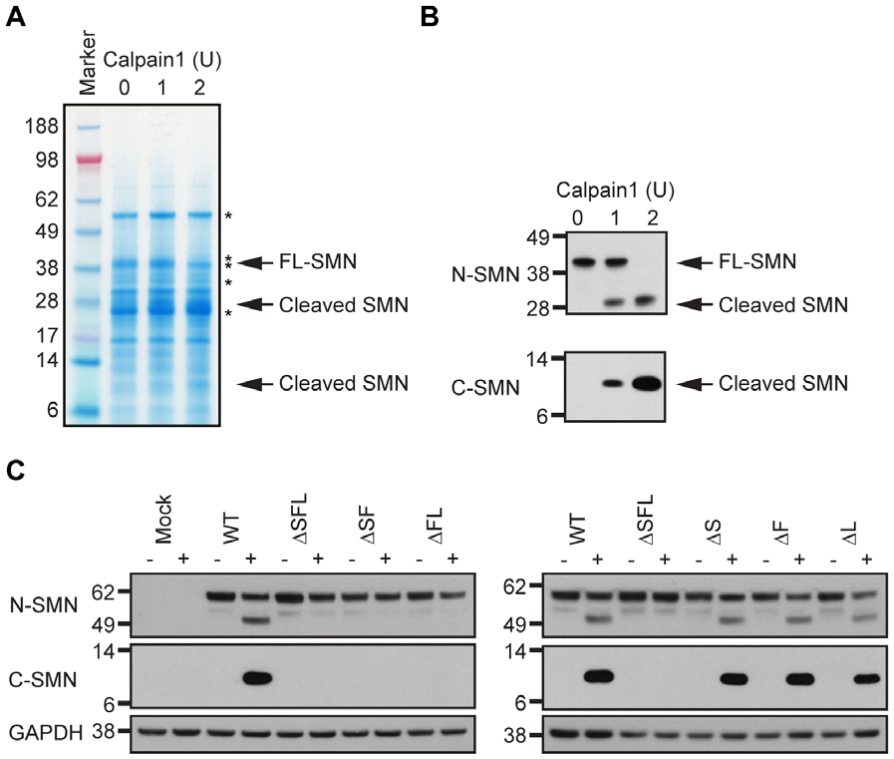
Mapping the calpain cleavage site. (A) Coomassie stained gel of HIS_6_-SMN/GST-Gemin2 heterodimers cleaved *in vitro* with indicated units of Calpain1 for 1 h. at 30°C. Full-length SMN (FL-SMN) as well as the N-terminal (N-SMN) and C-terminal (C-SMN) cleavage products are indicated with arrows. The C-terminal cleavage fragments were subjected to peptide fingerprint analysis. Asterisks (*) indicate full-length and truncated GST-Gemin2 proteins (see Fig. S2). (B) Western blot analysis of *in vitro* calpain assays. Antibodies recognizing the N- or C-terminus of SMN detected FL-SMN and SMN calpain cleavage products. The fraction of SMN cleavage was directly proportional to the amount of exogenous Calpain1 added. (C) Endogenous calpain cleavage assays were performed with EGFP-SMN containing small deletions within the calpain cleavage site. Double deletions blocked calpain cleavage, whereas single deletions did not.

Using mutational analysis, we successfully refined the CCR to amino acids 183-189 or 192-194, however it remained unclear whether these residues corresponded to the cleavage site or if they simply affected cleavage by another means. To precisely map the calpain cleavage site, we performed *in vitro* calpain cleavage reactions using purified recombinant HIS_6_-SMN/GST-Gemin2 heterodimers followed by mass spectrometric analysis of the C-terminal cleavage product. HIS_6_-SMN/GST-Gemin2 was co-expressed in *E. coli* and purified using glutathione sepharose beads. Gemin2 is a binding partner [22], [73] of SMN and was co-expressed to aid in SMN solubility in *E. coli* [74]. HIS_6_-SMN/GST-Gemin2 heterodimers were left either untreated, or were incubated with 1 mM CaCl_2_ and exogenous Calpain1 at 30°C. Cleavage products were analyzed on Coomassie stained SDS-PAGE gels and by Western blot. As previously demonstrated, Calpain1 cleavage of HIS_6_-SMN/GST-Gemin2 heterodimers produced the expected SMN cleavage fragments (Fig 3A,B and S2; [34]). The C-terminal cleavage product was excised, digested with trypsin, and the resultant peptides were subjected to MALDI MS/MS. Peptide fingerprint analysis identified nine different peptides in the calpain-treated samples (Table 1, Fig. S3, S4). As expected, no SMN peptides were obtained from the excised gel slice from the untreated sample. Among the nine peptides identified in the treated samples, three sequences (in italics) were represented, S_192_*FLPPPPP-MPGPR*L_205_, F_193_*LPPPPPMPGPR*L_205_, and R_204_*LGPGKPGLK*F_214_ (asterisks indicate cleavage sites). Trypsin cleaves after arginines and lysines. Therefore the first two peptides, which each have one non-tryptic end, indicate that calpain cleaves SMN after S192 or F193. The tryptic peptide, R_204_*LGPGKPGLK*F_214_, is immediately downstream of these peptides. Several other theoretical tryptic peptides were not detected due to their size. Only one expected tryptic peptide, R_288_*CSHSLN, was not identified. The mapped cleavage sites are in agreement with recognized amino acid preferences of calpain [60], [63] and several calpain cleavage prediction models, two of which predict F193 as the most probable calpain cleavage site in SMN. These models also predict S192 as a probable calpain cleavage site (see Calpain Modulatory Proteolysis Database, http://www.calpain.org/predict.rb?cls=substrate; [75]).

**Table 1:** Peptide fingerprint analysis of C-terminal SMN cleavage product.

Peptide Source	SMN Peptide Identified	Tryptic/Non-tryptic	Peptide m/z	MASCOT Ion Score
Untreated	None	NA	NA	NA
1U Calpain1	S_192_*FLPPPPPM_ox_PGPR*L_205_	Non-tryptic	1318.7067	9
	F_193_*LPPPPPMPGPR*L_205_	Non-tryptic	1155.6343	56
	F_193_*LPPPPPM_ox_PGPR*L_205_	Non-tryptic	1171.6345	24
	R_204_*LGPGKPGLK*F_214_	Tryptic	866.5449	40
2U Calpain1	S_192_*FLPPPPPMPGPR*L_205_	Non-tryptic	1302.7100	71
	S_192_*FLPPPPPM_ox_PGPR*L_205_	Non-tryptic	1318.7010	27
	F_193_*LPPPPPMPGPR*L_205_	Non-tryptic	1155.6340	65
	F_193_*LPPPPPM_ox_PGPR*L_205_	Non-tryptic	1171.6300	47
	R_204_*LGPGKPGLK*F_214_	Tryptic	866.5366	52

HIS_6_-SMN/GST-Gemin2 heterodimers were either untreated or cleaved with 1 or 2U of Calpain1. Bands containing the C-terminal calpain cleavage products were excised and subjected to peptide fingerprint analysis. An equivalent area in the untreated control sample was also analyzed. Nine peptides were identified (*italics*), four of which were in oxidized form (ox). Asterisks indicate the proteolytic cleavage sites. Non-tryptic peptides reveal the Calpain cleavage sites. Peptide m/z and MASCOT ion scores for each peptide are reported.

To further verify the results, we created double and single deletions within amino acids 192-194 in EGFP-SMN and assayed calpain susceptibility in endogenous calpain assays. Western analysis revealed that double deletions of S193,F193 (ΔSF) or F193,L194 (ΔFL) were capable of inhibiting calpain cleavage, whereas single deletions were not (Fig. 3C). Deletion of nearby residues (Δ177-182, Δ212-215) containing other putative calpain cleavage sites did not block SMN cleavage (data not shown). Altogether, these data reveal S192 and F193 as *bona fide* calpain cleavage sites.

### Removal of the SMN C-terminus, not SMN-oligomerization, affects calpain cleavage

The conserved YG box in SMN, with the aide of sequences corresponding to exon 2b, is important for the formation of SMN oligomers [47], [76], [77], [78], which are important for SMN complex formation and stability [13], [47]. SMA type I point mutations within the YG box (Y272C, G279V) disrupt this self-association, whereas SMA type II and III mutations (S262S, T274I), as well as SMNΔ7, show intermediate oligomerization defects [76]. The reduced stability of SMNΔ7 was recently proposed to be due to the presence of a degradation signal encoded by the YG box along with the residues EMLA [48], which are translated from exon 8 of the *SMN2* gene [79]. To determine if calpain cleavage could also play a role in SMN stability, we created mutations in the C-terminus of EGFP-SMN and assayed their susceptibility to endogenous calpains.

**Figure 4:**
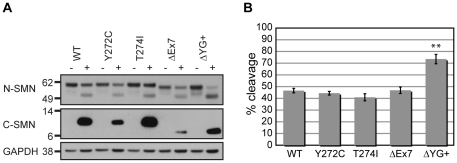
Deletion of the SMN C-terminus affects calpain cleavage. (A) A representative Western blot (for illustrative purposes only) demonstrating the calpain cleavage susceptibility of several C-terminal mutants. (B) Quantification of GFP-SMN cleavage was determined from Western blots probed with N-terminal SMN antibodies followed by Cy3 conjugated secondary antibodies (see Methods). The average fraction (expressed as %) of calpain cleavage was calculated from six independent cell-free cleavage assays. Removal of amino acids 268-294 (ΔYG+) in SMN renders it more susceptible to calpain cleavage, whereas mutations in the YG box or exon 7 (ΔEx7) did not. Error bars represent the SEM. Asterisks (**) indicate p value <0.001, determined by two-tailed Student T-test.

To quantify differences in calpain cleavage, we performed Western analysis using N-terminal SMN antibodies followed by Cy3-conjugated secondary antibodies. Using fluorometry, we quantified non-saturated signals from the full-length and the N-terminal EGFP-SMN calpain cleavage product. We calculated the average fraction of calpain cleavage observed for each mutant as compared to WT cleavage. To examine if SMN oligomerization defects perturbed calpain cleavage, we assayed two YG box mutant proteins, Y272C and T274I. We found that neither mutation affected calpain susceptibility, suggesting that monomeric SMN is not a better substrate for calpain cleavage (Fig. 4A,B). The calpain susceptibilities of SMNΔ7 and SMNΔ7+EMLA proteins were also similar to that of WT (Figs. 4A,B, and S5), indicating that calpain cleavage is not a primary contributor to the instability of SMNΔ7. Interestingly, we observed that deletion of the C-terminus, including the YG box (ΔYG+, Δ268-294), renders SMN more susceptible to calpain cleavage (Fig 4A,B). This increase suggests that the structure of the C-terminus affects the availability of the calpain cleavage site. In addition, removal of the SMN degron may not protect SMN from all proteases in the cell. Together, these results suggest that calpain is not a major determinant of SMN or SMNΔ7 stability in cultured cells.

### Patient-derived SMA mutations exhibit reduced calpain cleavage susceptibility

Although many SMA missense mutations reside in exons 6 and 7 of *SMN1*, others have been identified in exons 1-4. Characterization of a few of these mutant proteins has revealed that, although their ability to self-associate remains intact, they disrupt protein-protein interactions and can inhibit snRNP assembly [33]. We thus created various SMA mutations in EGFP-SMN and assayed their calpain cleavage susceptibility in endogenous calpain cleavage assays.

**Figure 5:**
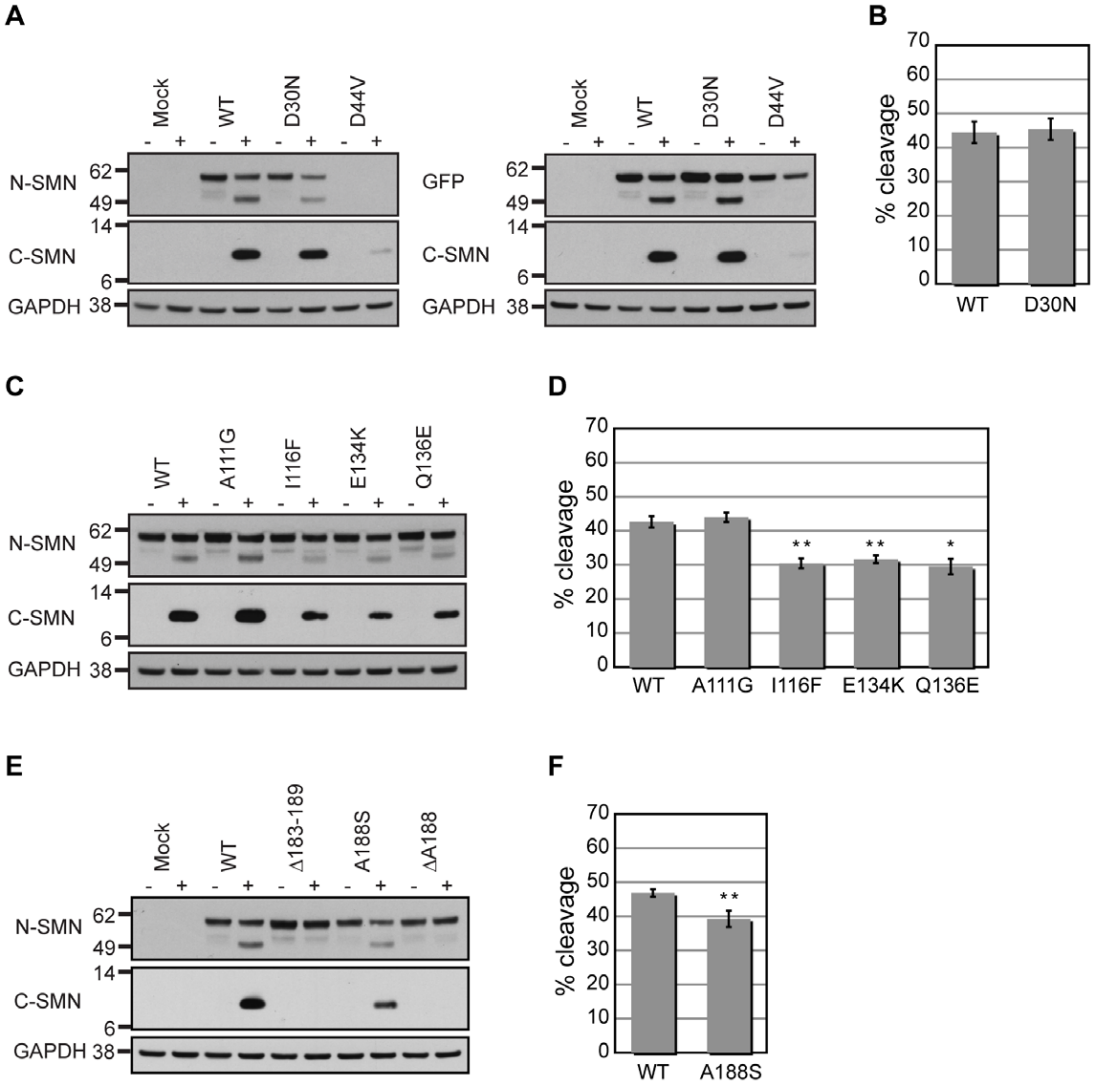
SMA mutations affect calpain cleavage. (A, C, E) Illustrative Western blot demonstrating the calpain cleavage susceptibility of several N-terminal mutants. (B, D, F) Quantification of GFP-SMN cleavage was determined from Western blots probed with N-terminal SMN antibodies followed by Cy3 conjugated secondary antibodies (see Methods). The average % of calpain cleavage was calculated from six independent cell-free cleavage assays. Error bars represent the SEM. Asterisks indicate p value, where p<0.005 (*) or p<0.001 (**), determined by two-tailed Student T-test. (A, B) Calpain cleavage of SMN(D30N) was similar to WT, whereas SMN(D44V) was drastically reduced, below the limits of quantification. Therefore, GFP antibodies were used only for detection of the EGFP-SMN(D44V) protein. (C, D) Three mutations within the Tudor domain (I116F, E134K, and Q146Q) showed slightly reduced susceptibility to calpain cleavage, whereas A111G behaved similar to WT. (E, F) Calpain cleavage of A188S was modestly reduced, but its deletion (ΔA188) greatly reduced calpain cleavage, below the limits of quantification.

The SMN-Gemin2 interaction is thought to provide a scaffold onto which other SMN complex components assemble [80], [81]. Gemin2 makes multiple contacts with SMN. It interacts with the region encoded by exon 2b [78] and the SMN-Gemin2 interaction is disrupted by competition with an N-terminal SMN peptide (aa13-44) or in the absence of the SMN N-terminus (Δ1-39) [73], [82]. Two SMA mutations in exon 2a show different characteristics. An SMA type II mutation (D30N) associates with WT SMN, interacts with Gemin2, and supports normal snRNP assembly, whereas these properties are disrupted by the SMA type III mutation SMN(D44V). Disruption of the SMN-Gemin2 interaction destabilizes the SMN complex. These effects are more pronounced when assayed in the backbone of the SMN exons1-5 truncation (SMN^ex1-5^) versus full-length SMN, presumably due to the absence of the C-terminal self-association domain [47], [83]. We assayed whether these mutations also had differences in calpain susceptibility using the cell-free system described above. Due to disruption of the epitope, the N-terminal anti-SMN antibody did not detect EGFP-SMN(D44V), so we used antibodies targeting GFP to examine cleavage of this mutant (Fig 5A). The EGFP-SMN(D30N) mutant showed similar cleavage susceptibility to WT, whereas the cleavage of D44V was dramatically inhibited (Fig. 5A,B). The results suggest that disruption of Gemin2 and/or SMN amino-terminal self-interactions alter the availability of the calpain cleavage site. When these mutations were assayed in the backbone of an EGFP-SMN^ex1-5^ truncation, the D44V mutation no longer blocked calpain cleavage, further supporting a role for the C-terminus in regulating availability of the cleavage site (Fig. S6).

The Tudor domain is a conserved motif found in several RNA binding proteins, including SMN [84]. The SMN Tudor domain is involved in binding several RG/RGG containing proteins, including the Sm proteins that are essential for snRNP assembly [66], [67], [68], [70]. Several mutations in the Tudor domain have been identified in SMA type I patients, three of which (I116F, E134K, and Q136E) were previously demonstrated to display reduced snRNP assembly activity [49]. We assayed calpain susceptibility of these mutations, along with the A111G mutation, which has normal snRNP assembly activity and moderate Sm protein and SMN association ability [49], [85]. We found A111G did not affect calpain susceptibility, whereas the I116F, E134K, and Q136E mutations showed slightly reduced calpain cleavage (Fig. 5C,D). Interestingly, the relative calpain susceptibility of the mutants shows the same trend as their relative snRNP assembly efficiencies. This raises the possibility that structural changes imposed by these mutations affect both snRNP assembly and calpain cleavage.

We demonstrated that EGFP-SMNΔ183-189, which neighbors the calpain cleavage site, was not cleaved by calpain (Figs. 2E, 5E). Previously, an SMA type I patient was found to have an A188S mutation in *SMN1* [86]. Although the A188S mutation slightly reduced calpain cleavage, deletion of this residue (Δ188) greatly impaired calpain cleavage, supporting the importance of this region for calpain cleavage (Fig. 5E,F). Together, the results demonstrate that certain SMA mutations can affect the calpain cleavage susceptibility of SMN.

### SMN is cleaved by cytoplasmic calpain

**Figure 6:**
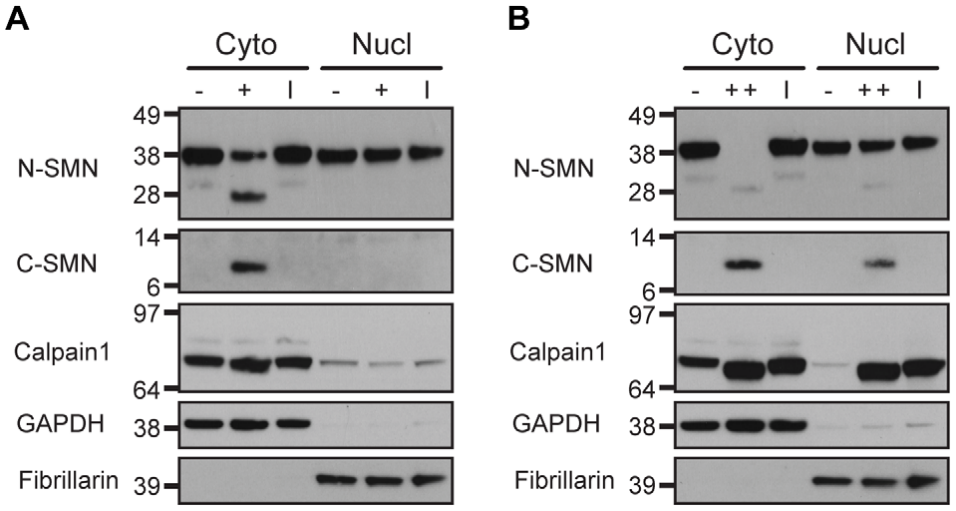
Endogenous calpain cleaves SMN in the cytoplasm. Cytoplasmic and nuclear extracts were prepared from U2-OS cells and used for cell-free calpain assays. Western blotting was performed to detect calpain cleavage of SMN, calpain activation, and fractionation efficiency. (A) Endogenous SMN was cleaved by endogenous calpain in the cytoplasm, but not in the nucleus. Extracts (30 µg) were left untreated (-), treated with 1 mM CaCl_2_ (+), or treated with calpain inhibitior (I) ALLN prior to CaCl_2_ addition. (B) Nuclear SMN was resistant to calpain cleavage even with the addition of exogenous Calpain1. Extracts (30 µg) were left untreated (-), treated with 1 mM CaCl_2_ and 1U of exogenous Calpain1 (++), or treated with calpain inhibitior (I) ALLN prior to addition of CaCl_2_ and Calpain1.

Calpains are considered to be cytoplasmic proteases; however there have been reports of several calpains that also localize to the nucleus [87], [88], [89], [90], [91]. Indeed, several nuclear proteins, including transcription factors, have been demonstrated as *in vitro* substrates of calpain. However, it is unclear if these proteins are cleaved in the nucleus or in the cytoplasm prior to import (reviewed in [37]). Like calpains, SMN also resides in the cytoplasm and nucleus [92], raising the question of the location of SMN cleavage. We therefore fractionated U2-OS cells into cytoplasmic and nuclear lysates prior to treatment with CaCl_2_, or with both CaCl_2_ and exogenous Calpain1. Western analysis showed that endogenous (Fig. 6A) and exogenous Calpain1 (Fig. 6B) readily cleaved SMN in the cytoplasm, whereas nuclear SMN was only cleaved upon addition of CaCl_2_ and exogenous Calpain1. Interestingly, the amount of nuclear cleavage was much less compared to cytoplasmic SMN cleavage, suggesting nuclear SMN is resistant to calpain. This resistance is not likely a result of experimental conditions, considering autolysis of Calpain1 also occurred in the nuclear fractions [93], as shown by a slight shift in the Calpain1 band size (Fig. 6A,B). Whether resistance is due to properties of nuclear SMN, or due to the activities of calpain and calpastatin within the nucleus is unknown. Considering the complete cleavage of cytoplasmic full-length SMN upon the addition of exogenous Calpain1 and CaCl_2_, the levels of the N-terminal cleavage product were lower than expected (Fig. 6B, lane 2). This may be the result of further cleavage and subsequent degradation of the N-terminal product, presumably due to the cell fractionation conditions and amount of exogenous Calpain1 present. Regardless, these results show that SMN is cleaved by endogenous calpains in the cytoplasm, consistent with the idea that calpain regulates only cytoplasmic functions of SMN.

## Discussion

We have previously shown that recombinant HIS_6_-SMN/GST-Gemin2 heterodimers and native SMN complexes are proteolytic targets of calpain [34]. To identify determinants of calpain cleavage, we analyzed the calpain susceptibility of mutant SMN proteins that contained internal deletions or SMA-derived point mutations. One identified determinant was the PEST motif, whose presence often indicates the existence of a nearby proteolytic cleavage site [64], [65]. Deletion of the PEST motif (aa133-174) in SMN blocks calpain cleavage, indicating that it is an important determinant of cleavage (Fig. 2B). However, the entire PEST motif is not necessary to direct calpain cleavage, as smaller deletions within this region do not block cleavage (Fig. 2C). Furthermore, deletion of the conserved Tudor domain (aa91-151), which partially overlaps with the PEST motif, does not block calpain cleavage (Fig. 2B). Interestingly, three SMA patient mutations within the Tudor domain (I116F, E134K, and Q136E), two of which also reside in the PEST motif, slightly impair calpain cleavage (Fig. 5C,D). These mutations are known to interfere with various protein interactions, such as with Sm proteins [66], [67], [68], [70], Fibrillarin [94], Gar1 [95], EBNA2 [96], hnRNPR [97], EWS [98], and KSRP/FBP2 [99]. Whether disruption of these or other interactions correlates with decreased calpain cleavage is currently unknown. It is possible that such interactions are necessary for SMN to maintain the optimal conformation for calpain cleavage. It is important to note that mutations (Y272C and SMNΔ7) that interfere with snRNP assembly do not affect calpain cleavage (Fig. 4A,B).

We found that residues within the N- and the C-termini of SMN are important for cleavage. Association of SMN with the SMN complex has been proposed to stabilize the protein [13], [47]. It was therefore of interest to determine if mutations that impair oligomerization increase the susceptibility to calpain. We found that such SMA patient mutations, Y272C, T274I, SMNΔ7, did not result in increased calpain susceptibility (Fig. 4A,B), suggesting calpain does not play a major role in the stability of SMN. This is consistent with the finding that treatment of cells with the calpain inhibitor calpeptin does not increase overall SMN levels [13], [48] and with observations that overexpression of calpastatin does not notably affect SMN levels or its localization in U2-OS (Fig. 2B and data not shown). These data, however, do not exclude the possibility that calpain might regulate a subpopulation of SMN proteins (e.g. within axonal or dendritic spines or other subdomains of the cytoplasm). Although removal of exon7 did not affect calpain cleavage of SMN, we showed that removal of the YG box along with exon7 (YG+), increased its susceptibility to calpain cleavage (Fig. 4A,B). This suggests that the C-terminus of SMN regulates the availability of the calpain cleavage site. Indeed, removal of the SMN degron does not protect SMN from calpain cleavage. It is noteworthy that the abundance of the C-terminal cleavage products for several of the C-terminal mutants does not reflect the levels of the corresponding N-terminal cleavage products (Figure 4). These results may indicate a difference in stability for these C-terminal cleavage products.

The importance of the C-terminus was also demonstrated by the ability of calpain to cleave EGFP-SMN^ex1-5^(D44V), but not the full-length mutant protein. Calpain cleavage was drastically impaired by the D44V mutation (Fig. 5A,B, S6). This mutation lies within exon 2a and has been found to impair Gemin2 binding [47]. Intriguingly, regions encoded by exons 2a and 2b, have been proposed to form intramolecular contacts with sequences encoded by exon 4 [78]. Thus, Gemin2, which is not itself a substrate for calpain when present the SMN complex [34], may be important for cleavage of SMN by calpain.

To map the calpain cleavage site, we implemented two approaches. The first monitored the calpain cleavage of numerous mutant proteins containing internal deletions within the CCR (Fig. 2). The second utilized peptide fingerprint mapping of the C-terminal cleavage product (Fig. 3A, 3B, S2). Identification of peptides containing non-tryptic termini revealed that calpain cleaves SMN after S192 or F193 (Table 1, Fig. S4, S5). Deletion of either residue does not block cleavage, however deletion of S192,F193 (ΔSF) or F193,L194 (ΔFL) blocked cleavage at these sites. These results support the peptide fingerprinting data (Fig. 3C). Interestingly, an uncharacterized SMA type I mutation, A188S, resides immediately upstream of the calpain cleavage site. This mutant protein is slightly less susceptible to calpain cleavage and its deletion blocks calpain cleavage (Fig. 5E,F), further demonstrating that this region of the protein is important for cleavage (Fig. 2E). Conversely, mutation of the prolines immediately downstream of the cleavage site did not significantly affect cleavage, suggesting there is some allowance for flexibility in neighboring residues (Fig. S1).

Cleavage of SMN results in two products, 1-193 and 194-294, fragments that contain the Tudor domain and proline-rich region, respectively (Fig. 2A). Localization of these fragments in U2-OS cells revealed a similar distribution to that of full-length SMN (data not shown). In contrast, similar SMN constructs (aa1-194 and aa190-294), were shown to have a pan-cellular localization in COS-1 cells, however, both constructs failed to localize to nuclear bodies [100]. It is possible that the differences in localization are a result of experimental conditions, including the cell lines and epitope tags used. It is currently unknown how calpain cleavage affects SMN function, however, it is tempting to speculate that calpain cleavage could lead to altered protein interactions or might regulate an activity of the SMN complex through incorporation of the cleavage products. Considering the large proportion of full-length SMN that remains after activation of endogenous calpains (Fig. 1A,B), it is likely that only a select population of SMN is cleaved.

Calpains are present in both the cytoplasm and the nucleus, however the vast majority of calpain substrates studied to date are cytosolic. Currently, it is unclear if reportedly nuclear substrates of calpain are actually cleaved in the nucleus following import [37]. The best characterized function of SMN is its role in the assembly of snRNPs in the cytoplasm, after which a fraction of SMN is thought to be imported into the nucleus, localizing within Cajal bodies and gems [101], [102]. We found that SMN is cleaved by endogenous calpains in the cytoplasm, but not in the nucleus (Fig. 6). Differences in calpain activity, SMN complex composition or differential post-translational modification, such as phosphorylation of nuclear SMN, might render it resistant to calpain cleavage. SMN is phosphorylated in the cytoplasm [103] and dephosphorylated in the nucleus by the nuclear phosphatase PPM1G [104]. In addition, protein kinase A (PKA) has been shown to phosphorylate SMN *in vitro*, at noncanonical sites [13]. How phosphorylation affects the calpain cleavage of SMN is unknown, however it is worth consideration since phosphorylation can affect susceptibility of calpain substrates and the activities of calpain and calpastatin (reviewed in [35], [37]).

The challenge now lies in determining how calpain might regulate SMN function *in vivo*. We previously demonstrated that depletion of SMN leads to both arborization defects and loss of myofibril integrity, even in the presence of normal snRNP levels, suggesting a tissue-specific function of SMN [26], [34]. SMN interacts genetically and physically with α-actinin [26], [105] and the SMN complex colocalizes with α-actinin at the myofibril Z-disc [26], [34]. Notably, over 20 muscle related diseases have been attributed to mutations in sarcomeric proteins, including Z-disc associated proteins, further underlining their importance [106]. Currently, the contribution of aberrant SMN muscle function to SMA is not fully understood. Interestingly, Z-disc associated SMN is a proteolytic target of calpain [34] however the fate of the cleavage products *in vivo* is unknown. Considering that several Z-disc proteins translocate from the sarcomere to the nucleus to perform signaling functions [106], it is conceivable that SMN and its cleavage products could have the same fate. Alternatively, it is possible that SMN calpain cleavage products are subsequently degraded by the proteasome. Indeed, sarcomeric proteins are only accessible to the proteasome following initial cleavage by other proteases such as caspases and calpains, indicating an important role for these upstream proteases during muscle remodeling [36], [107]. In addition to normal myofibrillar turnover, calpains are also implicated in muscle atrophy, as well as myogenesis [108]. Overexpression of calpastatin or depletion/inhibition of ubiquitous calpains impairs myoblast migration and fusion [109], [110], [111], [112], [113]. SMN has also been implicated in muscle regeneration. Low levels of SMN inhibit myoblast fusion [25], [114] and satellite cells expressing SMNΔ7 have limited regeneration potential [115]. Whether calpains contribute to the muscle atrophy seen in SMA patients by regulating a potential SMN muscle-related role is unknown, but is an attractive possibility.

Finally, calpain activity could be important for the proper functioning of SMN in neurons or may be affected as a result of SMA. SMN is associated with hnRNP-R, Zbp1, eEF1A, profilin II and β-actin mRNA and motoneurons isolated from severe SMA mice show a reduction of hnRNP-R, β-actin mRNA, and actin protein in the distal axons and growth cones, suggesting a role in local protein synthesis and actin dynamics in neurons (reviewed in [116]). SMA model mice display presynaptic and postsynaptic NMJ defects, such as impaired synaptic vesicle release, abnormal accumulation of presynaptic neurofilament protein (including phospho-isoforms), reduced AChR cluster size, and small myofibers [28], [117], [118], [119], [120]. In addition, a recent study demonstrated that TVA muscles from SMA mice (Smn^-/-^;SMN2;SMNΔ7) have increased (∼300%) asynchronous release frequency in the nerve terminals, unless assayed in the presence of EGTA. This suggests that there is a high increase in intraterminal bulk Ca^2+^ in these mice [120]. It is tempting to speculate that this increase in Ca^2+^ might affect the activity of calpains and consequently contribute to the SMA phenotype. Considering that calpain is involved in the dispersal of AChR clusters [121], [122] and that the dephosphorylated neurofilament protein is a calpain substrate [123], it is possible that altered calpain activity could lead to neurofilament accumulation and AChR cluster defects seen in SMA synapses. Furthermore, motoneurons isolated from severe SMA mice (*Smn^−/−^; SMN*2), or those depleted of hnRNP-R, show a reduction in clustering of N-type voltage-gated Ca^2+^ channels (Ca_v_2.2 channels), resulting in reduced Ca^2+^ transients in neuronal growth cones [124]. The potential effect on calpain activity is unknown, however it is noteworthy that, the Ca_v_β_3_ subunit of the Ca_v_2.2 channel is a calpain substrate and its proteolytic cleavage may be essential for regulation of the Ca^2+^ channel [125]. Overall, these observations suggest aberrant calcium signaling in the motoneurons of SMA mice. Such changes in calcium signaling could affect calpain activity and thereby the proteolytic regulation of a subpopulation of SMN in neurons.

Altogether, these relationships lead us to ask whether calpain cleavage of SMN is a physiologically productive event, leading to proper SMN function, versus a pathological result of aberrant calpain activation. Regardless, either scenario is interesting. In this study, we have laid the foundation for studying how calpain cleaves SMN by unveiling several determinants of cleavage. The next task at hand is to determine the physiological context under which calpain might play a role in regulating SMN function. This is a considerable challenge in view of the numerous putative functions for SMN and calpains, as well as the likelihood that calpain cleaves only a subpopulation of SMN in a context specific manner. However, the potential contribution by calpains to SMA pathology, indirectly, or directly through regulation of SMN, warrants the effort.

## Supporting Information

Figure S1 **Prolines in the CCR region do not affect calpain cleavage of SMN**. Deletion or substitutions of proline residues, P195-P199, were created in EGFP-SMN and transiently expressed in U2-OS cells. Endogenous calpain cleavage assays and subsequent Western analysis was performed to determine calpain cleavage susceptibility. Mutations of these proline residues within the CCR did not block calpain cleavage. Deletion of the prolines did reduce the size of the C-terminal cleavage product, suggesting the calpain cleavage site resides upstream of these residues.
(TIF)

Figure S2 **Identification of major protein bands present in the purified recombinant HIS_6_-SMN/GST-Gemin2 heterodimers preparation.** (A) Coomassie stained gel of HIS_6_-SMN/GST-Gemin2 heterodimers cleaved *in vitro* with indicated units of Calpain1 for 1 h. at 30°C. Full-length SMN (FL-SMN) as well as the N-terminal (N-SMN) and C-terminal (C-SMN) cleavage products are indicated with arrows. The C-terminal cleavage fragments were subjected to peptide fingerprint analysis. Asterisks (*) indicate full-length and truncated GST-Gemin2 proteins (see Fig. 3). (B) Western blot analysis of *in vitro* calpain assays. Antibodies recognizing the N- or C-terminus of SMN detected FL-SMN and SMN calpain cleavage products. The fraction of SMN cleavage was directly proportional to the amount of exogenous Calpain1 added. Antibodies recognizing Gemin2 or GST detected full-length and truncated GST-Gemin2 proteins.
(TIF)

Figure S3 **MS/MS spectra obtained from the C-terminal calpain cleavage product of SMN.** Recombinant HIS_6_-SMN/GST-Gemin2 heterodimers were treated with 1U of Calpain1, subjected to reduction and alkylation, and resolved on a Coomassie stained SDS-PAGE gel. The C-terminal calpain cleavage product was excised from the gel, typsinized, and the resultant peptides were analyzed by MALDI TOF/TOF mass spectrometry. Four peptides (A-D) were matched to SMN by peptide mass and MS/MS fragmentation. (A) S_192_*FLPPPPPM_ox_PGPR*L_205_, m/z  = 1318.7067 (B) F_193_*LPPPPPMPGPR*L_205_, m/z  = 1155.6343 (C) F_193_*LPPPPPM_ox_PGPR*L_205_, m/z  = 1171.6345 (D) R_204_*LGPGKPGLK*F_214_, m/z  = 866.5449. Two peptides (A, C) were in oxidized form (ox). Asterisks indicate the proteolytic sites. Non-tryptic peptides (A-C) reveal the calpain cleavage sites. Peptide m/z for each peptide is reported.
(TIF)

Figure S4 **MS/MS spectra obtained from the C-terminal calpain cleavage product of SMN.** Recombinant HIS_6_-SMN/GST-Gemin2 heterodimers were treated with 2U of Calpain1, subjected to reduction and alkylation, and resolved on a Coomassie stained SDS-PAGE gel. The C-terminal calpain cleavage product was excised from the gel, typsinized, and the resultant peptides were were analyzed by MALDI TOF/TOF mass spectrometry. Five peptides (A-E) were matched to SMN1 by peptide mass and MS/MS fragmentation. (A) S_192_*FLPPPPPMPGPR*L_205_, m/z  = 1302.7100 (B) S_192_*FLPPPPPM_ox_PGPR*L_205_, m/z  = 1318.7010 (C) F_193_*LPPPPPMPGPR*L_205_, m/z  = 1155.6340 (D) F_193_*LPPPPPM_ox_PGPR*L_205_, m/z  = 1171.6300 (E) R_204_*LGPGKPGLK*F_214,_ m/z  = 866.5366. Two peptides (B, D) were in oxidized form (ox). Asterisks indicate the proteolysis sites. Non-tryptic peptides (A–D) reveal the calpain cleavage sites. Peptide m/z for each peptide is reported.
(TIF)

Figure S5 **Calpain susceptibility of SMNΔ7 and SMNΔ7+EMLA.** Mutations were created in EGFP-SMN and transiently expressed in U2-OS cells. Endogenous calpain cleavage assays and subsequent Western analysis were performed to determine calpain cleavage susceptibility. No obvious difference in calpain cleavage was seen between SMNΔ7 and SMNΔ7+EMLA.
(TIF)

Figure S6 **Calpain susceptibility of D30N and D44V mutations in SMN^ex1-5^.** Mutations were created in EGFP-SMN^ex1-5^ and transiently expressed in U2-OS cells. Calpain cleavage of full-length WT EGFP-SMN was assayed in parallel. Endogenous calpain cleavage assays and subsequent Western analyses were performed to determine calpain cleavage susceptibility. (A) Antibodies recognizing the N-terminus of SMN detected WT SMN proteins. (B) Anti-GFP antibodies were used to detect the D44V mutant protein. Results show that both WT and mutant proteins were cleaved by calpain and produced similar N-terminal cleavage products. These results suggest that the C-terminus is important for availability of the calpain cleavage site. Furthermore, the data show that the cleavage site is located within residues encoded by exons 1-5. Asterisk (*) indicates an additional calpain cleavage product observed for the EGFP-SMN^ex1-5^ truncation (WT and D44V). This additional calpain cleavage product was only observed using anti-GFP antibodies. Cleavage products observed in the untreated lysate suggest that the EGFP-SMN^ex1-5^ protein is susceptible to additional proteases (unrelated to calpain).
(TIF)
