# Supplementary figures and images for: Molecular Determinants of Survival Motor Neuron (SMN) Protein Cleavage by the Calcium-Activated Protease, Calpain
Source: PLoS One. 2010 Dec 30;5(12):e15769. doi: 10.1371/journal.pone.0015769 (PMC3012718; doi:10.1371/journal.pone.0015769)

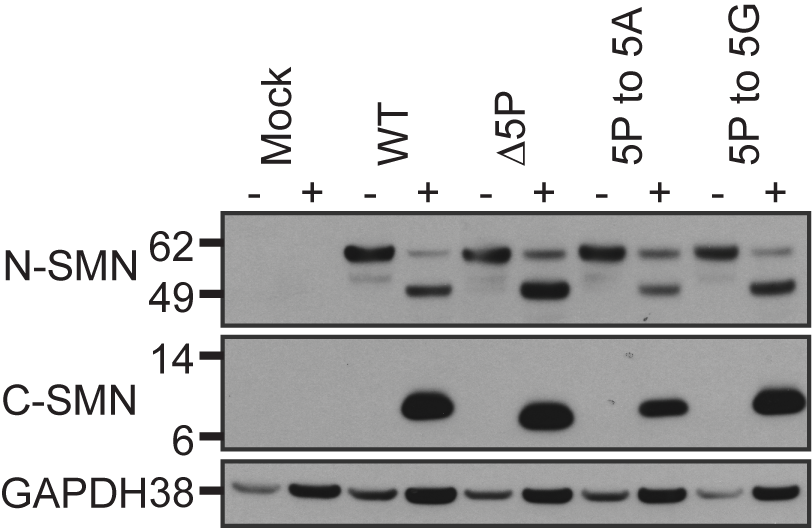

Supplement: Figure S1 — Prolines in the CCR region do not affect calpain cleavage of SMN. Deletion or substitutions of proline residues, P195-P199, were created in EGFP-SMN and transiently expressed in U2-OS cells. Endogenous calpain cleavage assays and subsequent Western analysis was performed to determine calpain cleavage susceptibility. Mutations of these proline residues within the CCR did not block calpain cleavage. Deletion of the prolines did reduce the size of the C-terminal cleavage product, suggesting the calpain cleavage site resides upstream of these residues. (TIF) [file pone.0015769.s001.tif]

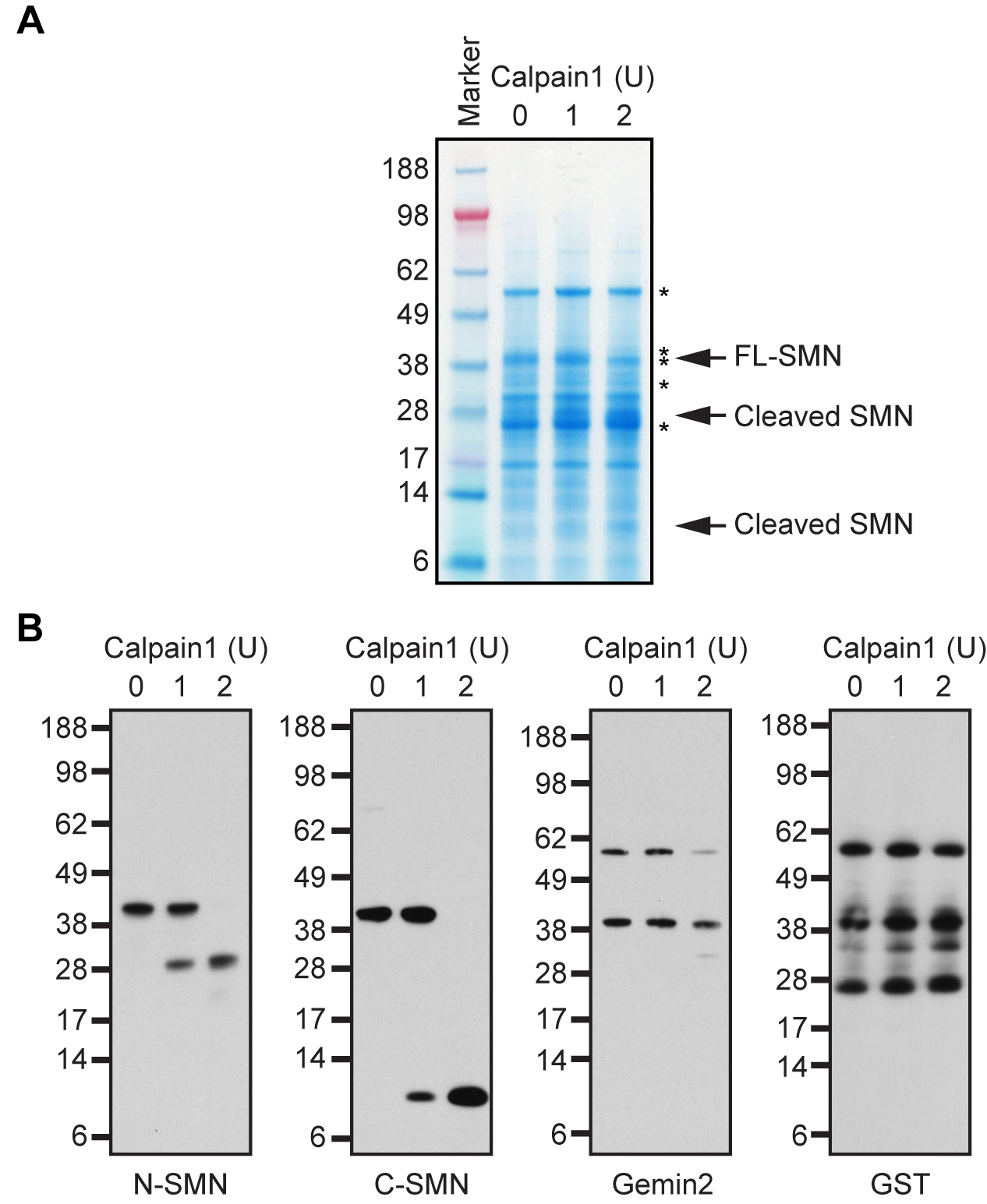

Supplement: Figure S2 — Identification of major protein bands present in the purified recombinant HIS6-SMN/GST-Gemin2 heterodimers preparation. (A) Coomassie stained gel of HIS6-SMN/GST-Gemin2 heterodimers cleaved in vitro with indicated units of Calpain1 for 1 h. at 30°C. Full-length SMN (FL-SMN) as well as the N-terminal (N-SMN) and C-terminal (C-SMN) cleavage products are indicated with arrows. The C-terminal cleavage fragments were subjected to peptide fingerprint analysis. Asterisks (*) indicate full-length and truncated GST-Gemin2 proteins (see Fig. 3). (B) Western blot analysis of in vitro calpain assays. Antibodies recognizing the N- or C-terminus of SMN detected FL-SMN and SMN calpain cleavage products. The fraction of SMN cleavage was directly proportional to the amount of exogenous Calpain1 added. Antibodies recognizing Gemin2 or GST detected full-length and truncated GST-Gemin2 proteins. (TIF) [file pone.0015769.s002.tif]

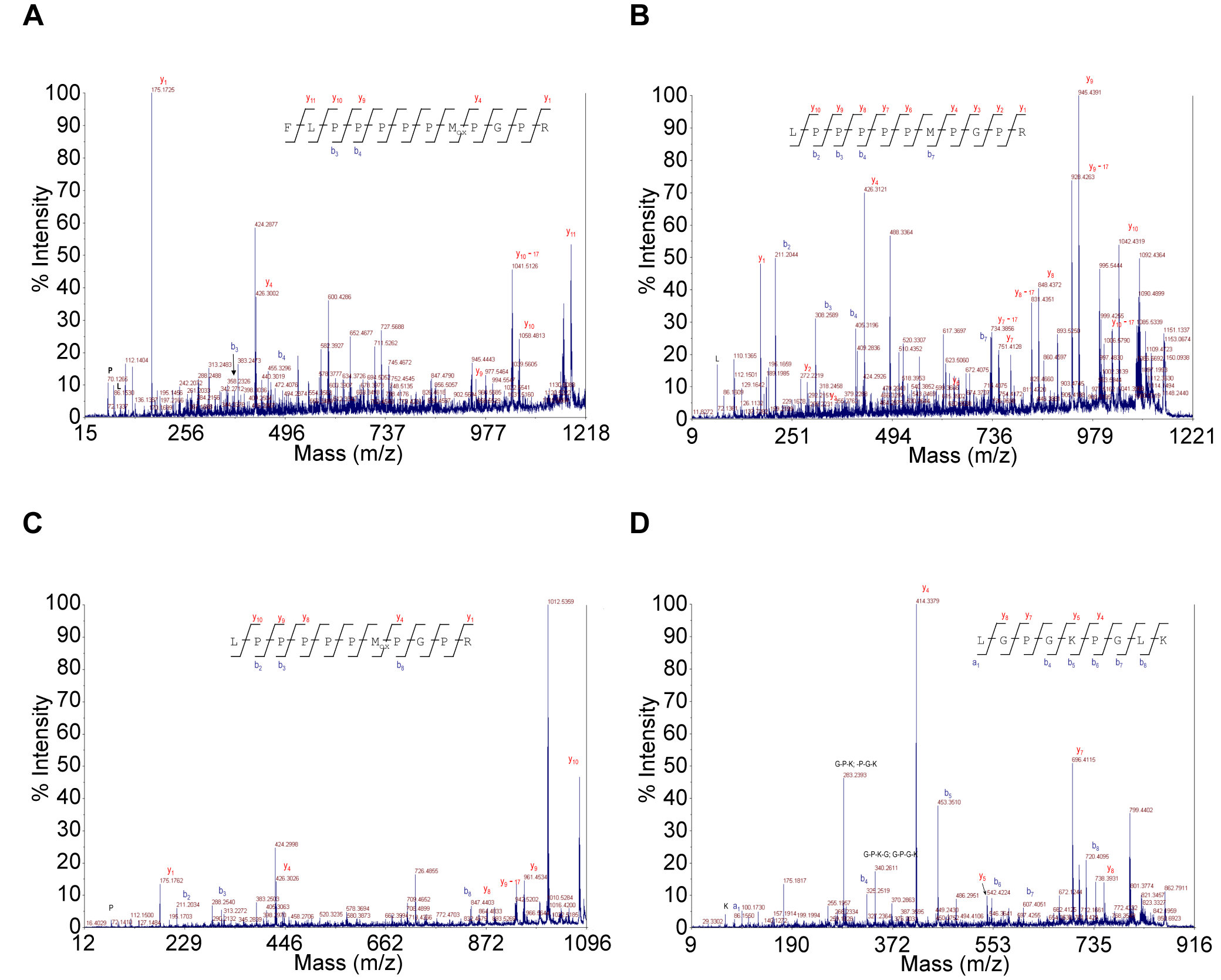

Supplement: Figure S3 — MS/MS spectra obtained from the C-terminal calpain cleavage product of SMN. Recombinant HIS6-SMN/GST-Gemin2 heterodimers were treated with 1U of Calpain1, subjected to reduction and alkylation, and resolved on a Coomassie stained SDS-PAGE gel. The C-terminal calpain cleavage product was excised from the gel, typsinized, and the resultant peptides were analyzed by MALDI TOF/TOF mass spectrometry. Four peptides (A-D) were matched to SMN by peptide mass and MS/MS fragmentation. (A) S192*FLPPPPPMoxPGPR*L205, m/z = 1318.7067 (B) F193*LPPPPPMPGPR*L205, m/z = 1155.6343 (C) F193*LPPPPPMoxPGPR*L205, m/z = 1171.6345 (D) R204*LGPGKPGLK*F214, m/z = 866.5449. Two peptides (A, C) were in oxidized form (ox). Asterisks indicate the proteolytic sites. Non-tryptic peptides (A-C) reveal the calpain cleavage sites. Peptide m/z for each peptide is reported. (TIF) [file pone.0015769.s003.tif]

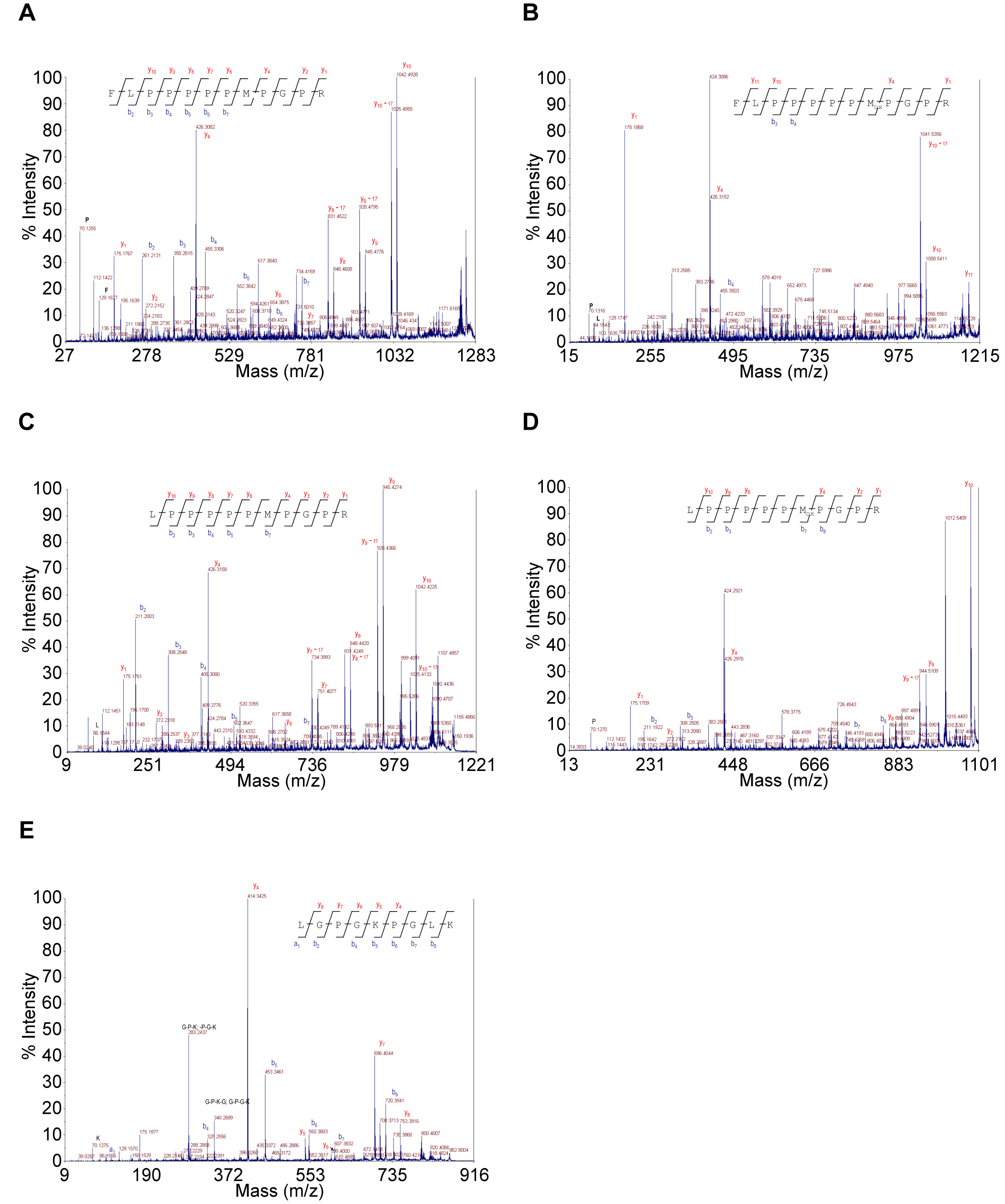

Supplement: Figure S4 — MS/MS spectra obtained from the C-terminal calpain cleavage product of SMN. Recombinant HIS6-SMN/GST-Gemin2 heterodimers were treated with 2U of Calpain1, subjected to reduction and alkylation, and resolved on a Coomassie stained SDS-PAGE gel. The C-terminal calpain cleavage product was excised from the gel, typsinized, and the resultant peptides were were analyzed by MALDI TOF/TOF mass spectrometry. Five peptides (A-E) were matched to SMN1 by peptide mass and MS/MS fragmentation. (A) S192*FLPPPPPMPGPR*L205, m/z = 1302.7100 (B) S192*FLPPPPPMoxPGPR*L205, m/z = 1318.7010 (C) F193*LPPPPPMPGPR*L205, m/z = 1155.6340 (D) F193*LPPPPPMoxPGPR*L205, m/z = 1171.6300 (E) R204*LGPGKPGLK*F214, m/z = 866.5366. Two peptides (B, D) were in oxidized form (ox). Asterisks indicate the proteolysis sites. Non-tryptic peptides (A–D) reveal the calpain cleavage sites. Peptide m/z for each peptide is reported. (TIF) [file pone.0015769.s004.tif]

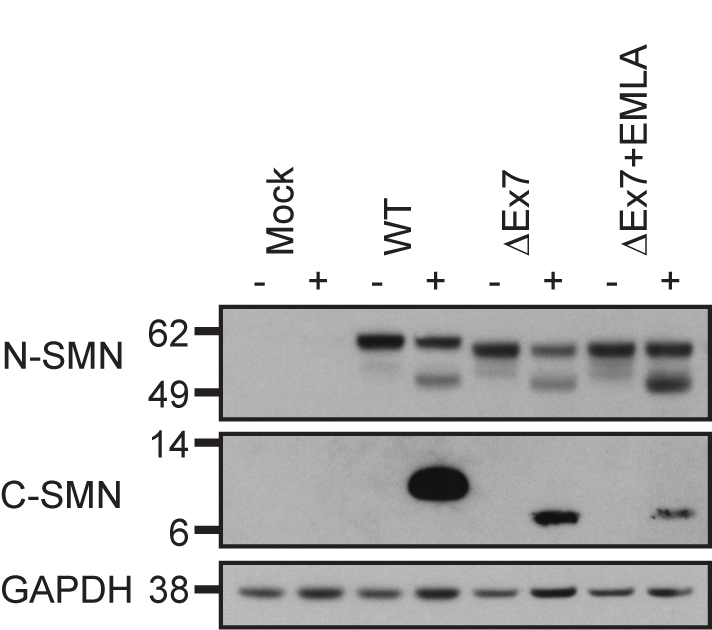

Supplement: Figure S5 — Calpain susceptibility of SMNΔ7 and SMNΔ7+EMLA. Mutations were created in EGFP-SMN and transiently expressed in U2-OS cells. Endogenous calpain cleavage assays and subsequent Western analysis were performed to determine calpain cleavage susceptibility. No obvious difference in calpain cleavage was seen between SMNΔ7 and SMNΔ7+EMLA. (TIF) [file pone.0015769.s005.tif]

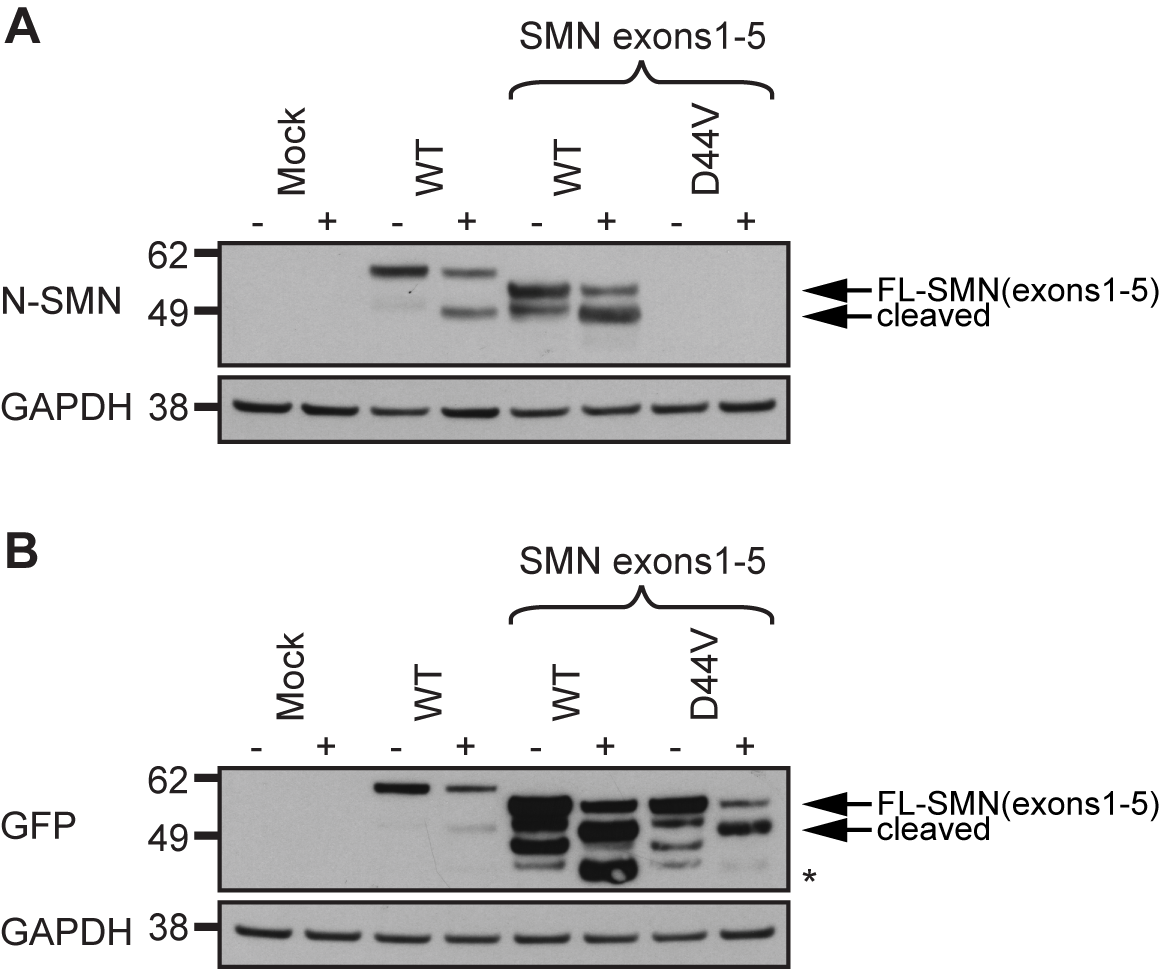

Supplement: Figure S6 — Calpain susceptibility of D30N and D44V mutations in SMNex1-5. Mutations were created in EGFP-SMNex1-5 and transiently expressed in U2-OS cells. Calpain cleavage of full-length WT EGFP-SMN was assayed in parallel. Endogenous calpain cleavage assays and subsequent Western analyses were performed to determine calpain cleavage susceptibility. (A) Antibodies recognizing the N-terminus of SMN detected WT SMN proteins. (B) Anti-GFP antibodies were used to detect the D44V mutant protein. Results show that both WT and mutant proteins were cleaved by calpain and produced similar N-terminal cleavage products. These results suggest that the C-terminus is important for availability of the calpain cleavage site. Furthermore, the data show that the cleavage site is located within residues encoded by exons 1-5. Asterisk (*) indicates an additional calpain cleavage product observed for the EGFP-SMNex1-5 truncation (WT and D44V). This additional calpain cleavage product was only observed using anti-GFP antibodies. Cleavage products observed in the untreated lysate suggest that the EGFP-SMNex1-5 protein is susceptible to additional proteases (unrelated to calpain). (TIF) [file pone.0015769.s006.tif]
